# Editorial: Perspectives in clinical microbiology for combating multi-drug resistant bacterial infections: 2024/2025

**DOI:** 10.3389/fcimb.2026.1783280

**Published:** 2026-01-23

**Authors:** Mithun Rudrapal, Teresa Estrada-Garcia

**Affiliations:** 1 Department of Pharmaceutical Sciences, School of Biotechnology and Pharmaceutical Sciences, Vignan’s Foundation for Science, Technology and Research, Guntur, India; 2 Center for Research and Advanced Studies, National Polytechnic Institute of Mexico (CINVESTAV), México City, Mexico

**Keywords:** bacterial infections, clinical microbiology, drug discovery, ESKAPE pathogens, hospital-acquired infections, multi-drug resistance

The global escalation of antimicrobial resistance (AMR) including antibiotic resistance represents a critical threat to public health, driven by the overuse and misuse of antimicrobial drugs and antibiotics in clinical, veterinary, and agricultural settings. The rapid emergence of multidrug-resistant (MDR) bacterial pathogens has rendered many existing antimicrobial treatment options ineffective, leading to persistent and recurrent infections that are difficult to cure. Such bacterial pathogens include Gram-negative bacteria including ESKAPE (*Enterococcus faecium*, *Staphylococcus aureus*, *Klebsiella pneumoniae*, *Acinetobacter baumannii*, *Pseudomonas aeruginosa* and *Enterobacter* spp) and other opportunistic bacteria (El-Sayed et al., 2025; Yang et al., 2025). These pathogens are major causes of life-threatening hospital-acquired infections (HAIs) such as urinary tract infections (UTIs), pneumonia, surgical site infections, bloodstream infections (BSIs) or sepsis, and meningitis resulting in high morbidity, mortality, and economic burden worldwide (Sannathimmappa et al., 2025). MDR bacterial pathogens employ diverse mechanisms to evade antimicrobial action, including altered cell membrane permeability, efflux pump over expression, biofilm formation, enzymatic drug inactivation, and modifications of drug targets. Globally, MDR infections contribute to over 700,000 deaths annually, with projections estimating up to 10 million deaths per year by 2050 if resistance continues to rise unchecked. The economic burden is similarly staggering; with estimates suggesting costs of up to USD 100 trillion by 2050 due to prolonged hospital stays, higher treatment expenses, and increased mortality (Macesic et al., 2025). Advances in modern rapid diagnostics and newly approved antibacterial drugs including antibiotics have improved detection and treatment, but these MDR bacterial pathogens continue to challenge clinical management increasing treatment failure worldwide. In response to this challenge, innovative anti-infective strategies are being explored to counter emergence and spread of MDR bacterial pathogens. Promising therapeutic approaches include antimicrobial peptides, anti-virulence compounds, quorum-sensing inhibitors, bacteriophage therapy, photodynamic therapy, stem cell therapy, immunotherapeutics and novel natural therapeutics (Elshobary et al., 2025). Further, drug repurposing, CRISPR-Cas genetic interventions, and nanobiotics/nanoparticles offer alternative avenues to enhance treatment efficacy (He et al., 2025). Additionally, artificial intelligence offers a promising tool to accelerate pathogen detection and the discovery and optimization of effective antibacterial interventions towards combating MDR infections and mitigate the global burden of antimicrobial resistance.

The Research Topic *“Perspectives in Clinical Microbiology for Combating Multi-drug Resistant Bacterial Infections 2024/2025”* was aimed to compile latest research ideas, reviews and perspectives focusing on the theme of the topic within the scope of the journal. This series shed light on contemporary and emerging perspectives in Clinical Microbiology research towards potential developments in Drug Discovery approaches by fostering a diverse range of scholarly contributions. The topic was led by two Guest Editors listed above who are experts in the subject and oversaw the entire editorial process for the submitted papers. A total of fourteen articles were published, including eleven original research, two review and one mini-review articles.

Cantas et al. reports that effective antibiotic therapy significantly suppresses the expression of resistance plasmid transfer genes and mitigates inflammatory responses *in vivo*, whereas ineffective or sub-inhibitory antibiotic treatment activates bacterial SOS responses and enhances ARG dissemination. These findings provide direct *in vivo* evidence that inappropriate antibiotic selection or dosing can accelerate the spread of antibiotic resistance genes and worsen disease outcomes, underscoring the critical importance of optimized antimicrobial use within a One Health framework.

Chen et al. demonstrates that bloodstream infections caused by *Acinetobacter baumannii* are associated with high mortality, particularly those due to carbapenem-resistant strains belonging to ST2 and KL2/3/7/77/160 lineages. Increased mortality was linked to severe clinical conditions, extensive prior antibiotic exposure, invasive procedures, and hypoalbuminemia rather than marked differences in virulence phenotypes. The predominance of CRAb carrying bla_OXA-23_ and bla_OXA-66_, along with enrichment of virulence genes related to iron acquisition and biofilm formation, underscores the importance of early molecular typing and targeted clinical management to improve outcomes in patients with *A. baumannii* bloodstream infections.

A study by Demirhan et al. reports that newly synthesized unsymmetrical imidazolium salts exhibit strong acetylcholinesterase inhibitory activity and notable antimicrobial effects, particularly against Gram-negative bacteria. The combined *in vitro* and *in silico* results support their dual bioactivity and highlight these compounds as promising candidates for the development of alternative antimicrobial agents and acetylcholinesterase inhibitors.

In another study, Guo et al. claims that antibiotic-loaded bone cement (ALBC) combined with negative pressure wound therapy is more effective than conventional negative pressure wound therapy (NPWT) alone in treating multidrug-resistant diabetic foot ulcers. The combined approach significantly reduced inflammation, improved local vascular and healing indicators, accelerated bacterial clearance and wound healing, and shortened hospital stay, indicating that ALBC plus NPWT is a promising therapeutic strategy for managing MDR organism-related diabetic foot ulcers.

Kong et al. identifies key clinical and treatment-related risk factors associated with carbapenem-resistant *Enterobacterales* bloodstream infection (CRE-BSI), including prior exposure to broad-spectrum antibiotics, glucocorticoid use, surgical history, and invasive procedures. CRE-BSI was associated with markedly high in-hospital mortality, with arterial catheterization emerging as an independent predictor of death. Advanced age, underlying respiratory and digestive diseases, and infection type significantly influenced 30-day survival. These findings highlight the need for targeted risk stratification, judicious antibiotic use, and careful management of invasive interventions to reduce the burden and mortality of CRE-BSI.

Ma et al. establishes a novel nanoluciferase-based reporter phage (φFN) that enables rapid, sensitive, and accurate drug susceptibility testing of *Mycobacterium tuberculosis* for all first-line antitubercular drugs within 72 hours. The φFN assay demonstrated high sensitivity and specificity compared with the MGIT 960 system, with luminescence signals correlating well with MIC values. These findings highlight φFN as a promising rapid diagnostic tool for early detection of drug-resistant TB, offering significant advantages in turnaround time and clinical applicability.

A real-world multicenter study conducted by Qin et al. repots that ceftazidime–avibactam (CVA) is widely used and clinically effective for the treatment of multidrug-resistant Gram-negative infections in China, particularly those caused by carbapenem-resistant *Klebsiella pneumoniae*. Despite high rates of meropenem resistance and carbapenemase gene carriage, resistance to CVA remained low. Treatment with CVA resulted in favorable clinical cure and microbiological success rates, supporting its role as an important therapeutic option for severe, drug-resistant infections in routine clinical practice.

In a study, Song et al. characterizes a multidrug-resistant *Klebsiella michiganensis* strain (LS81) producing bla_KPC-2_ on a transferable IncM2 plasmid (pLS81-KPC), confirming its potential for horizontal gene transfer to other bacteria. Whole-genome analysis revealed sequence type ST196, multiple β-lactamase genes, and integration of bla_KPC-2_ within a truncated Tn6296 transposon, highlighting the strain’s multidrug resistance and genomic plasticity. Phylogenetic and pan-genomic analyses emphasize the strain’s close relation to global clinical isolates and underscore the urgent need for ongoing surveillance of *K. michiganensis* as an emerging hospital-acquired pathogen.

Taotao et al. demonstrates that class 1 integrons are highly prevalent among clinical *Aeromonas hydrophila* isolates and play a central role in mediating multidrug resistance through the carriage of resistance gene cassettes, particularly aadA1 and catB8. Integron-positive isolates showed strong associations with corresponding antibiotic resistance phenotypes and exhibited high clonal similarity, indicating potential intra-hospital dissemination, especially in hepatobiliary surgery wards. These findings highlight the value of integron profiling as an effective molecular tool for surveillance and targeted infection control of multidrug-resistant *A. hydrophila*.

Wang et al. reports that bile microbiota composition varies according to the etiology of biliary obstruction, with *Klebsiella pneumoniae*, *Acinetobacter baumannii* and *Escherichia coli* as the predominant bacterial species. Next-generation sequencing showed a higher detection rate than conventional culture and enabled broader identification of bacterial and fungal communities in bile. These findings support the utility of NGS as an effective complementary tool for characterizing bile microbiota and improving the microbiological assessment of biliary obstructive diseases.

Yin et al. establishes a robust and clinically useful nomogram for predicting perioperative multidrug-resistant bacterial and fungal infections in patients with gastrointestinal fistulas. Multidrug-resistant infections were predominantly caused by Gram-negative bacilli, particularly carbapenem-resistant *Enterobacteriaceae*. Secondary surgery and perioperative inflammatory markers were identified as key risk factors. The model demonstrated strong predictive performance and clinical utility, supporting early risk stratification and targeted infection prevention strategies in this high-risk population.

In a review, Ohia et al. demonstrates that a coordinated One Health approach is essential for addressing multidrug-resistant bacterial infections, as AMR arises from interconnected human, animal, and environmental systems. Evidences from multiple countries show that regulatory reforms, integrated surveillance, community engagement, and emerging AI-driven diagnostics can significantly improve MDR control. However, persistent challenges including governance fragmentation, limited laboratory capacity, ethical concerns, and inequitable access to AI technologies in LMICs must be addressed. Sustained investment in intersectoral collaboration, equitable innovation, and community empowerment is critical to reducing the global MDR burden and strengthening health system resilience.

In a short review, Oliveira et al. emphasizes that multidrug-resistant bacterial infections pose a critical global health challenge driven by widespread antibiotic misuse, rapid dissemination of resistance mechanisms, and limitations of conventional diagnostics. The integration of rapid diagnostic tools and artificial intelligence, alongside the development of innovative therapeutic strategies such as bacteriophages, antimicrobial peptides, and microbiome-based approaches, offers promising solutions. Effective control of antimicrobial resistance ultimately depends on rational antibiotic use, sustained research investment, and coordinated global multidisciplinary collaboration.

In another review, Wang et al. highlights that integrating MALDI-TOF MS with machine learning offers a promising, rapid, and cost-effective approach for predicting antimicrobial resistance. Large-scale, well-curated datasets and optimized algorithms including hyperparameter tuning and ensemble learning can significantly improve predictive performance and robustness, with AUROC emerging as a key evaluation metric. Model interpretability further enhances clinical acceptance. However, challenges such as limited sample sizes, lack of data standardization, and constrained generalizability remain. Continued methodological optimization and standardization are essential for translating MALDI-TOF MS-ML approaches into reliable tools for routine AMR surveillance and clinical decision-making.

In conclusion, this Research Topic underscores the critical advances and ongoing challenges in addressing multidrug-resistant infections. By integrating original research, reviews, and expert perspectives, this Research Topic highlights novel insights into antimicrobial resistance mechanisms, surveillance strategies, and innovative drug discovery approaches. Collectively, these contributions emphasize the importance of continued multidisciplinary research, collaboration, and translation of findings into clinical practice to effectively combat the global threat of multidrug-resistant including antibiotic-resistant bacterial infections. Further, multidisciplinary strategies embedded in the series may provide a roadmap for early detection, targeted therapy, mitigating antibiotic resistance, guiding future research, and improving global health outcomes in the face of an escalating antimicrobial crisis.
